# Early Stage of Atherosclerosis in Aortocoronary Saphenous Vein Grafts: Intravascular Ultrasound Study

**DOI:** 10.21470/1678-9741-2018-0221

**Published:** 2019

**Authors:** Przemysław Węglarz, Tomasz Bochenek, Grzegorz Bajor, Katarzyna Mizia-Stec, Michał Krejca, Maria Trusz-Gluza

**Affiliations:** 1 First Department of Cardiology, Medical University of Silesia, Katowice, Poland.; 2 Department of Human Anatomy, Medical University of Silesia, Katowice, Poland.; 3 Department of Cardiac Surgery, Medical University of łódź, Lodz, Poland.

**Keywords:** CABG, Venous Grafts, Prostheses, Atherosclerosis

## Abstract

**Introduction:**

Angiographically visible plaques in patent vein grafts are usually detected years after surgery. Our aim was to examine early plaque formation in vein grafts.

**Methods:**

Bypass angiography and intravascular ultrasonography (IVUS) examination were performed on 77 aortocoronary saphenous vein grafts (SVGs) implanted in 36 patients during the first 2 years after CABG. In each graft, a good quality 25 mm ultrasound image was analyzed. We measured: plaque area, lumen area, external elastic membrane (EEM) area, graft area and wall area. For the comparative assessment of SVGs, the index plaque area/EEM area was calculated. Data were analyzed for the following 4 time periods: I – 0-4 months (22 grafts), II – 5-8 months (23 grafts), III – 9-12 months (19 grafts) and IV – 13-16 months (13 grafts) after CABG. Student’s t and Fisher-Snedecor tests were used for the purpose of statistical analysis in this retrospective study.

**Results:**

In period I, plaque formation (neointimal) was observed in 10 grafts (45%), with a mean plaque area of 1.59 mm., in 6 grafts (26%) in period II, with a mean plaque area of 1.03 mm. and in 15 grafts (71%) in period III, with a mean plaque area of 1.41 mm., and in all (100%) grafts in period IV, with mean plaque area of 2,3 mm.. Average index plaque area/EEM area in periods I, II, III and IV were 0.12, 0.08, 0.13 and 0.22. We have showed a significant plaque increase between periods II and IV(*P*=0.038).

**Conclusion:**

IVUS showed plaque in about 40% of venous grafts during the first year after CABG. Between 13-16 months plaque was visible in all studied grafts.

**Table:** 

Abbreviations, acronyms & symbols	
CABG	= Coronary artery bypass grafting
EEM	= External elastic membrane
IVUS	= Intravascular ultrasonography
QCU-CMS	= Quantitative coronary ultrasound-clinical measurement solution
RCA	= Right coronary artery
SVG	= Saphenous vein graft

## INTRODUCTION

The long-term benefits of an internal thoracic artery graft are well established and remain the gold standard for revascularization^[1]^. Data on long-term outcomes of arterial revascularization in venous grafts continue to show better effects even in the most recent literature^[2]^. Nevertheless, because it is not always possible to achieve complete revascularization through only arterial grafts, the use of saphenous vein grafts (SVGs) is still common worldwide.

It has been shown that venous grafting induces inflammation and endothelial cell damage and dysfunction, which promote migration and proliferation of vascular smooth cells^[3]^.

A significant limitation of SVGs is their patency^[4]^. Therefore, the correct identification of the neointimal formation in venous grafts may help in the decision-making regarding the intensification of lipid-lowering therapy, the use of antiplatelet drugs or even gene therapy in the future. The aim of these therapies is to slow or preferably reverse the progression of plaque formation within grafted veins. During the first year, there are about 15% of occlusions in implanted aortocoronary venous bypasses^[4-6]^. It is estimated that ten years after surgery, only 60% of implanted aortocoronary venous bypasses remain patent and only 50% of patent bypasses are free of atheromatous formations^[7,8]^. Despite the common treatment of ischemic heart disease using aortocoronary venous bypasses, there are a few publications available concerning the *in vivo* observation of neointimal formation in SVGs in patients. The aim of the study was to analyze the formation of neointima in venous grafts in the first 1.5 years after the surgical procedure using intravascular ultrasonography (IVUS). In a study by Willard et al.^[9]^, there was a good correlation between the ultrasound imaging and histological analysis, which enabled the normal intima, intimal hyperplasia, venous wall fibrosis and atheromatous plaque to be distinguished. One study that used virtual histology showed that the severity of SVG atherosclerosis was parallel with a proportional increase in fibrofatty tissue^[10]^.

## METHODS

### Patients

Our study was a subanalysis of a study, already published, that concerned externally stented saphenous vein grafts. In the following study, only subgroup of non-stented grafts were analyzed. It was approved by the local Ethics Committee of the Medical University of Silesia in Katowice. The Bioethics Commission number for approval of our study was L.dz. NN-013-267/01. All patients provided informed consent.

The inclusion criteria for our main study were prior to CABG and included: men and women, age 40-65 years, with multivessel coronary disease, critical stenosis in right coronary artery (RCA), stable angina, systolic blood pressure below 160 mmHg, blood glucose below 7.8 mmol/L. The exclusion criteria were: lack of written consent, inability to perform complete arterial revascularisation, varicose veins, poor saphenous vein quality, unstable angina, low ejection fraction (LVEF <30%), concomitant valve disease, critical carotid artery stenosis, Leriche syndrome, and any other condition that limited life expectancy to less than 2 years.

Seventy-seven separate IVUS examinations of venous bypasses were analyzed in 36 patients. IVUS assessment started in the first patient in the first month following CABG and ended in the last patient 16 months after surgery. Every patient was assessed only once after bypass surgery.

The evaluation of the plaque, including IVUS follow-up studies, was divided into four time periods: I – 0-4 months (22 grafts), II – 5-8 months (23 grafts), III – 9-12 months (19 grafts) and IV – 13-16 months (13 grafts) after CABG. The patient details are presented in Table 1. Different times of admissions in the Department of Cardiac Surgery were a reason for the different follow-up of our patients. The Department first cared for patients who needed urgent care and therefore the study patients were sometimes unintentionally delayed. This has been the reason why a different time assessment finally took place. The final analysis should depend on these deadlines.

**Table 1:** Patient’s clinical characteristics.

Age (years)	55±8
Hypertension (>140/90 mmHg)	69%
Dyslipidemia (total cholesterol >200 mg or LDL >135 mg%)	61%
Smoking	45%
Diabetes mellitus	0%
Previous cardiac arrest	50%

### Coronary Angiography and Intravascular Ultrasonography

The study was conducted in the Department of Invasive Cardiology at the Medical University of Silesia in Katowice, Poland.

All the patients who were analyzed underwent angiography of the native coronary vessels, followed by selective angiography of the venous bypasses.

IVUS examinations were performed on a Volcano Therapeutics Inc. (CA, USA) system using the 20 MHz ultrasonography "eagle-eye" probes with the “pull-back” device moving the probe at a rate of 1 mm/s. The examination was preceded by direct administration of 5000 IU of heparin and 0.2 mg of nitroglycerin to the SVG.

**Fig. 1:**
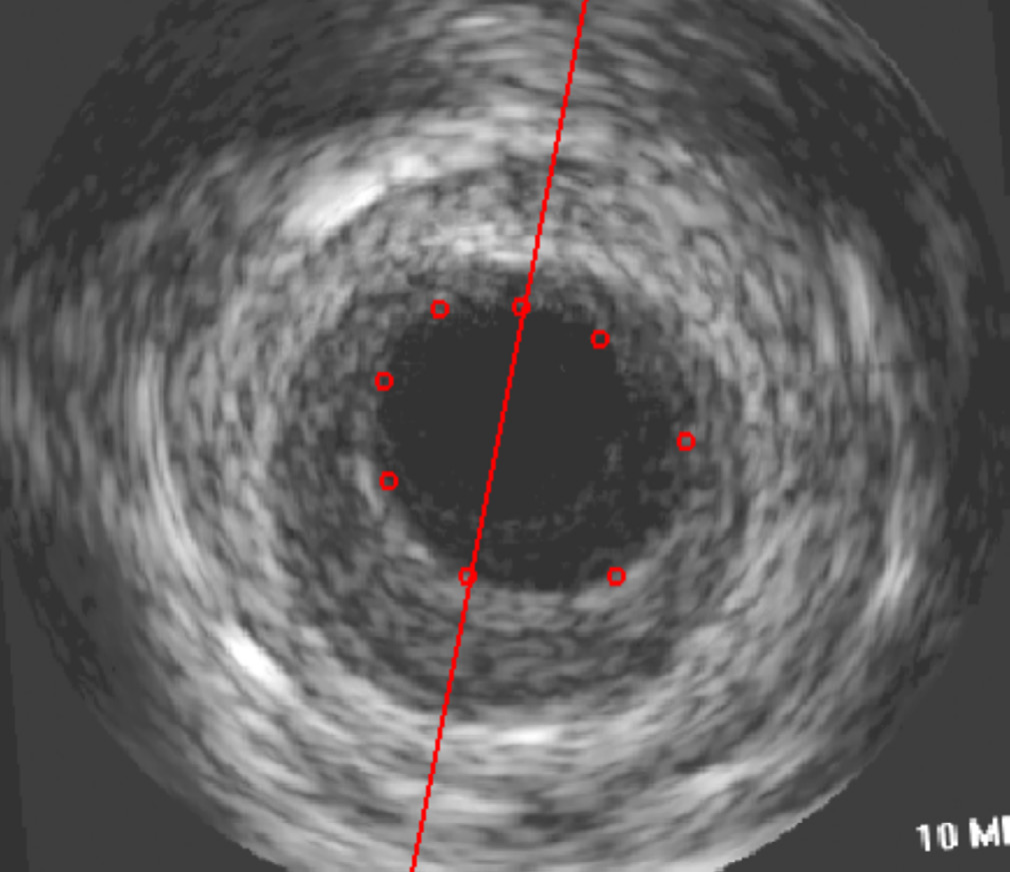
Analysis of venous bypass grafts using QCU-CMS IVUS analytical software.

The analysis of IVUS examinations was performed using Quantitative Coronary Ultrasound-Clinical Measurement Solution (QCU-CMS) IVUS software, which was adjusted to analyze venous bypasses (Figure 1).

The analyzed sections consisted of 250 single ultrasound images (10 individual “pictures” were estimated by 1 mm). The venous bypass section was selected based on image quality, including the area of the bypass that had the most advanced degree of neointimal formation.

**Fig. 2:**
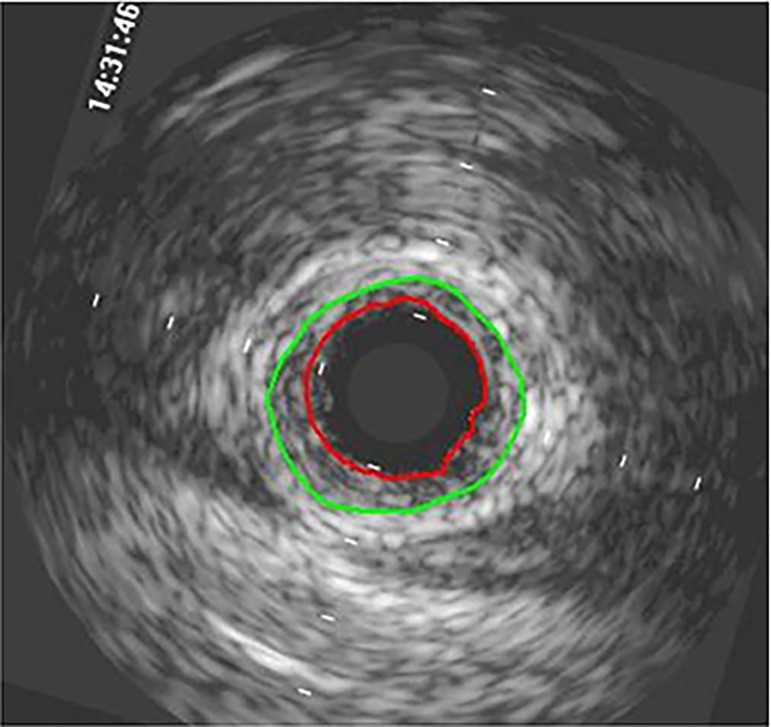
Delineation of neointima (red line) and external elastic lamina (green line) in a saphenous vein bypass graft.

We excluded sections of venous bypasses that were directly adjacent to the proximal and distal sections of the bypass from the analysis. In the examinations, all images that had an unequivocally visible echo-negative limit were included (Figure 2). We measured the lumen area, the external elastic membrane (EEM) area (measured by tracing the outer border of the sonolucent zone), the SVG area (measured by tracing the outer border of the entire venous graft) and the wall area (defined as the SVG area minus the EEM area). The plaque area (defined as the difference between the EEM area and the lumen area), the SVG wall area (defined as the difference between the outer border of the entire venous graft area and the EEM area) and the plaque area/EEM area index were automatically calculated to perform a comparative assessment of the SVGs.

### Statistical Analysis

The results of the measurements for the specific time periods are presented as the arithmetical mean (±) and standard deviation. The Student’s t-test for equal or unequal variances of the analyzed variables was used to analyze the importance of the differences between the means. At the same time, an analysis of the importance of the differences between the means, coupled with an analysis of the equality of the variances, was determined using Fisher-Snedecor’s F statistics. We adopted P<0,05 as significant for our results.

## RESULTS

The mean age of our group was 55±8 years old. Most of our patients had hypertension and dyslipidaemia, and almost half the study group were smokers. We did not include patients with diabetes mellitus. Fifty percent of our group was previously diagnosed with myocardial infarction.

**Fig. 3:**
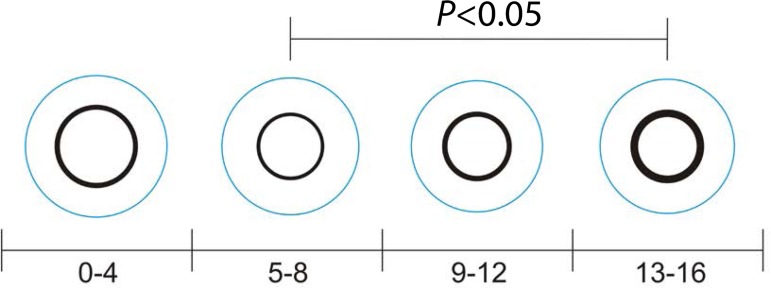
Neointimal formation in the four time periods analysed.

The angiographic and IVUS examination, performed 64 days after the surgical procedure, indicated the first presence of neointima formation. Table 2 shows the results of the measurements performed during the ultrasound examinations. In the first period, plaque (neointimal) formation was observed in ten grafts (45%), with a mean plaque area of 1.59 mm^2^; in period II, six grafts (26%) had a mean plaque area of 1.03 mm^2^; in period III, 15 grafts (71%) had a mean plaque area of 1.41 mm^2^, while in period IV, all of the grafts (100%) had a mean plaque area of 2.3 mm^2^. We observed a reduction in the EEM area between periods I and II. We showed a significant plaque increase between periods II and IV (*P*=0,041) (Figure 3) The plaque area/EEM area index in the fourth analyzed period differed significantly compared to the first studied periods, which is also shown in the table.

**Table 2:** Results of the measurements performed during IVUS examinations.

Observation period	0-4 months	5-8 months	9-12 months	13-16 months
Lumen area of SVG (mm^2^)	10.92±39	9.14±3.0	8.90±3.0	8.80±3.0
EEM area (mm^2^)	12.52±3.9	* 10.17±3.9 *P* =0.050	10.45±3.0	11.07±2.5
Plaque area (mm^2^)	1.58±2.2	1.02±2.1	1.41±1.7	2.30±1.0
Plaque area/EEM area index	0.12±0.1	0.08±0.1	0.13±0.1	*0.22±0.1 *P* =0.038
SVG area (mm^2^)	21.26±4.9	20.58±6.2	19.69±5.2	20.14±3.6
SVG wall area (mm^2^)	8.57±2.7	10.41±3.9	9.24±2.9	9.08±2.1

* Values for which *P* ≤0.05 in proportion to the period of 0 to 4 months. EEM=external elastic membrane; SVG=saphenous vein graft

## DISCUSSION

The 20^th^ century brought very rapid developments in all fields of medicine. Advances in cardiac surgery and cardiology directly reflect the results of treatments and the life span of patients. The development of treatment techniques, as well as diagnostic methods specifically in cardiology, progressed simultaneously. In the case of invasive cardiology, intravascular ultrasound imaging of vessels, as well as other imaging modalities, is currently in a strong position and, in most interventional cardiology centers, it is applied as common procedure in clinical studies and everyday medical practice. The method discussed significantly dominates other angiographic examinations, especially for the diagnosis of early stages of atheromatous plaque development.

Two-dimensional image of venous bypasses in angiographic examination and only the evaluation of vessel lumen substantially limits the application of this method to assess the processes involved in venous bypasses, including evaluation of atheromatous plaque formation^[12,13]^.

The advantage of intravascular ultrasound examination is the possibility of visualizing the entire cross-section of both aortic and venous vessels and measuring their specific elements, *i.e*., size, wall thickness, plaque value, etc^[12]^. The aforementioned parameters enable a very detailed analysis of plaque development and the adaptive mechanisms involved in the vessel^[12,14]^. The interpretation of intravascular ultrasound relies on simple visual inspection of acoustic reflections to determine plaque composition^[15]^.

In our study, the external elastic membrane (EEM) in the intravascular examinations of coronary arteries was usually measured as the inner limit of the zone of hypoechogenicity that represented the media^[6,7]^. In contrast to the arteries, evaluation of EEM in the case of venous bypasses is much more difficult. This is because remodelling processes, including proliferation of myofibroblasts and formation of extracellular substances, affect the entire vessel cross-sectional area. The replacement of the adventitia and media by connective tissue results in difficulties in the evaluation of EEM, even in a microscopic examination^[11]^.

Higucki compared IVUS examinations performed on 15 venous bypasses up to the first month after the procedure and 14 bypasses imaged 6 to 12 months after the procedure. His analysis showed that, between the analyzed periods, the vessel’s walls thicken significantly and its lumen was preserved, and this thickening was compensated for the enlargement of the entire bypass. The lumen of a venous bypass had a decreasing tendency between the two periods analysed, but this was not statistically significant^[14]^.

The accuracy of the typical atheromatous plaque that was evaluated in the IVUS examinations was estimated for a period of about nine months after the operation^[16]^. which was much earlier than the previous angiographic examinations showed.

In Hozumie’s comparative analysis of venous bypasses performed along with an IVUS examination conducted in the 1^st^ month after the procedure, as well as bypasses that had been subjected to an IVUS examination within 6 to 15 months after the surgical procedure, a substantial increase of intima value was found^[13]^.

In his studies, Tashihiko performed intravascular ultrasonography examinations in venous bypasses within a period of 8 to 23 years after CABG^[17]^. This study did not confirm the suggestion that veins are susceptible to compensate for the enlargement in the site of growing atheromatous plaque. It is highly probable that severe fibrosis occurs within the adventitia and inhibits the ability for a vessel to adapt. Based on the examination performed, we conclude that the lumen of venous bypasses decreases in the first six months after the surgical procedure, after which it maintains a similar value in further observation in a statistically significant manner.

Although the mean values of the cross-sectional area of the formatting atheromatous plaque tended to increase, statistically significant differences were not found.

The mean values of EEM did not show an increasing trend over time. A statistically significant decrease in the EEM mean cross-sectional value of a venous bypass was only found between the first and second periods.

The results of our study indicate that there is an intense process of neointimal formation within the first 18 months after the procedure, which at this time includes most of the venous bypasses. A decrease in the lumen value in venous bypasses is most probably part of processes such as the development of neointima, without compensating for vessel thickening, which is probably related to the intense fibrosis that occurs within adventitia during this period.

Thus, the formatting neointima provides the basis for venous bypass atherogenesis in the future. Furthermore, the intense process of its formation may lead to venous bypass occlusion. Taking into account the fact that the process of formation of the atheromatous plaque of neointima in venous bypasses occurs early, it is very important to counteract this process by taking actions to minimize any venous damage during the surgical procedure and to exclude any risk factors^[18,19]^.

### Limitations of the Method

The relatively low number of patients is a limitation of our study. IVUS analysis was performed in 25 mm sections of aortocoronary venous bypass. This section has a very good image quality, as well as the visible formation of neointima. However, the entire venous bypass was analyzed, which may limit the comprehensiveness of the conclusions.

## CONCLUSION

The IVUS showed *in vivo* plaque formation in about 40% of aortocoronary venous grafts during the first year after CABG. Between 13 and 16 months, plaque was visible in all grafts.

Despite the rapid development of interventional cardiology, CABG still plays an important role in everyday practice and remains a treatment option for significant number of patients; therefore, studies concerning potential improvements of this method, as well as the follow-up treatment, are still required.

**Table:** 

Authors’ roles & responsibilities	
PW	Contributed equally to the study in acquisition, analysis and interpretation, as well as writing of the manuscript; final approval of the version to be published
TB	Contributed equally to the study in acquisition, analysis and interpretation, as well as writing of the manuscript; final approval of the version to be published
GB	Analysis interpretation, as well as revising the manuscript; final approval of the version to be published
KMS	Analysis interpretation, as well as revising the manuscript; final approval of the version to be published
MK	Analysis interpretation, as well as revising the manuscript; final approval of the version to be published
MTG	Analysis interpretation, as well as revising the manuscript; final approval of the version to be published
